# Heroin inhibits HIV-restriction miRNAs and enhances HIV infection of macrophages

**DOI:** 10.3389/fmicb.2015.01230

**Published:** 2015-11-04

**Authors:** Xu Wang, Tong-Cui Ma, Jie-Liang Li, Yu Zhou, Ellen B. Geller, Martin W. Adler, Jin-Song Peng, Wang Zhou, Dun-Jin Zhou, Wen-Zhe Ho

**Affiliations:** ^1^School of Basic Medical Sciences, Wuhan UniversityWuhan, China; ^2^Department of Pathology and Laboratory Medicine, Lewis Katz School of Medicine, Temple University, PhiladelphiaPA, USA; ^3^The Center for Animal Experiment/ABSL-III Laboratory, Wuhan UniversityWuhan, China; ^4^Center for Substance Abuse Research, Lewis Katz School of Medicine, Temple University, PhiladelphiaPA, USA; ^5^Wuhan Center for Disease Prevention and ControlWuhan, China

**Keywords:** Heroin, HIV, IFN-α/β, miRNAs, macrophage

## Abstract

Although opioids have been extensively studied for their impact on the immune system, limited information is available about the specific actions of opioids on intracellular antiviral innate immunity against HIV infection. Thus, we investigated whether heroin, one of the most abused drugs, inhibits the expression of intracellular HIV restriction microRNA (miRNA) and facilitates HIV replication in macrophages. Heroin treatment of macrophages enhanced HIV replication, which was associated with the downregulation of several HIV restriction miRNAs. These heroin-mediated actions on the miRNAs and HIV could be antagonized by naltrexone, an opioid receptor antagonist. Furthermore, the *in vitro* negative impact of heroin on HIV-associated miRNAs was confirmed by the *in vivo* observation that heroin addicts had significantly lower levels of macrophage-derived HIV restriction miRNAs than those in the control subjects. These *in vitro* and *in vivo* findings indicate that heroin use compromises intracellular anti-HIV innate immunity, providing a favorable microenvironment for HIV survival in the target cells.

## Introduction

Injection drug users (IDUs) now represent one of the largest reservoirs of HIV infection in the United States, contributing to the fastest spread of the virus (Alcabes and Friedland, 1995; Risdahl et al., 1998). IDUs frequently use heroin, the most commonly abused opiate (Martin et al., 2010). Opiate use is associated with the worst health outcomes in HIV-infected individuals, as they adversely impact the human immune system. Clinical and epidemiological evidence from pre-AIDS studies indicate that opiates (particular heroin) play a cofactor role in the pathogenesis of HIV infection (Donahoe et al., 1993; Ronald et al., 1994; Specter, 1994; Meijerink et al., 2015; Zhou et al., 2015). *In vitro* studies from different laboratories also provide direct evidence that opiates (morphine) facilitate HIV infection of the target cells. Morphine enhanced HIV replication in human monocytes/macrophages (Guo et al., 2002; Ho et al., 2003; Li et al., 2003; Wang et al., 2011a), T lymphocytes (Chuang et al., 1993; Steele et al., 2003; Peterson et al., 2004), kupffer cells (Schweitzer et al., 1991), human neuroblastoma cells (Squinto et al., 1990), and human brain cells (Chao et al., 1995; Peterson et al., 1999; Reynolds et al., 2006).

The enhancing effect of opiates on HIV is likely due to their negative impact on the host immune defense mechanisms. Opiates are known to have a profound effect on the immune system (Wang and Ho, 2011; Wang et al., 2011b; Samikkannu et al., 2015). To date, the immunosuppressive effects of opiates have been extensively investigated in the major cell types of the immune system, including natural killer cells, T cells, B cells, macrophages and polymorphonuclear leukocytes (Brack et al., 2011; Eisenstein, 2011; Boland et al., 2014; Pomorska et al., 2014). Macrophages, as a primary target of HIV infection, are among the first cells infected by HIV and late function as a reservoir for the virus. Although opioids have been shown to modulate the function of macrophages, there is limited information about the specific actions of opiates, particularly heroin, on intracellular antiviral innate immunity that controls HIV replication in macrophages. Earlier studies reported that morphine suppressed the production of type I interferons (IFNs; Peterson et al., 1989; Wang et al., 2002), the key cytokines that modulate all phases of immune processes and play a central role in host innate immunity against viral infections. In response to viral infections, IFN-α/β can trigger down-stream cell signaling and subsequent induction of many IFN-stimulated genes (ISGs) and other antiviral factor. IFN-α/β also induce antiviral miRNAs (Pedersen et al., 2007; Zhou et al., 2010; Cobos Jimenez et al., 2012). Studies have demonstrated that the miRNAs participate in the host immune responses to viral infections, including HIV (Huang et al., 2007; Wang et al., 2009; Kumar, 2011). In general, the miRNAs interfere with HIV replication by either directly binding to viral RNAs or targeting the cellular factors that are related to HIV survival (Huang et al., 2007; Klase et al., 2012; Chen et al., 2014). Several cellular miRNAs (miR-28, 29a, 125b, 150, 198, 223, and 382) have been identified to target a set of accessory genes of HIV (Hariharan et al., 2005; Ahluwalia et al., 2008; Nathans et al., 2009; Sung and Rice, 2009; Wang et al., 2009). For example, these HIV restriction miRNAs can target the 3′UTR of HIV transcripts, potentially rendering productive infection of HIV into latency in resting CD4^+^ T lymphocytes (Huang et al., 2007). We reported that monocytes from peripheral blood are enriched with some of these HIV restriction miRNA, which contribute to the resistance of monocytes to HIV infection (Wang et al., 2009). Given the key role of the HIV restriction miRNAs in intracellular innate immunity, it is of significance to determine whether environmental factors such as heroin abuse can dysregulate these miRNAs in the target cells of HIV.

## Materials and Methods

### Study Subjects

Fourteen heroin addicts and eight control subjects were recruited by the Wuhan Center for Disease Prevention and Control (Wuhan CDC) in China. Informed consent was obtained from the study subjects, and the Institutional Research Board of the Wuhan CDC approved this study. The majority (>80%) of heroin users in China use heroin only (Lu et al., 2008). Polydrug use was excluded based on the self-report and Urine Screen. All the subjects were current heroin users as their urine tests were positive for opiate at the time of enrollment. The control subjects were recruited using convenience sampling from the community in which the study site was located. Subjects were excluded if they had a chronic systemic illness (cardiac, renal, pulmonary, hepatic, endocrine, metabolic, or autoimmune disorders), major psychiatric disorders or if they were abusing other substances other than heroin (urine drug test). Control subjects with no history of drug or alcohol abuse were also excluded if they had major medical or psychiatric disorders. All the study subjects were negative for HIV and HCV.

### Cell Isolation and Culture

Peripheral blood samples were obtained from healthy adult donors without a known history of drug abuse, and identified as HIV-1 antibody negative. Monocytes were purified as per the previously described technique (Wang et al., 2009, 2011a). In brief, heparinized blood is separated by centrifugation over Lymphocyte Separation Medium at 400–500X*g* for 45 min. The mononuclear layer is collected and incubated with Dulbecco’s Modified Eagle’s Medium (DMEM) in 2% gelatin-coated flasks for 45 min at 37°C, followed by removal of the non-adherent cells with DMEM. Following the initial purification, at least 97% of the cells are monocytes, as determined by flow cytometry analysis using a monoclonal antibody against CD14, a marker specific for monocytes and macrophages. Purified monocytes are plated in 48-well culture plates (0.25 × 10^6^ cells/well) or 96-well culture plates (10^5^ cells/well) in DMEM containing 10% fetal calf serum (FCS) for 7 days. Monocyte-derived macrophages (MDMs) refer to 7-days-cultured macrophages.

### RNA Extraction and Real-time RT-PCR

Total cellular RNA, including miRNA, was extracted from the cells using the miRNeasy Mini Kit from QIAGEN (Valencia, CA, USA). Total RNA (1μg) was reverse-transcribed with the miScript Reverse Transcription Kit from QIAGEN. Real-time RT PCR for the quantification of a subset of miRNAs (miRNA-28, miRNA-125b, miRNA-150, miRNA-382, miRNA-223, miRNA-122, Let-7c, and miRNA-124a) was carried out with miScript Primer Assays and the miScript SYBR Green PCR Kit from QIAGEN as described (Wang et al., 2009). For mRNA detection, the resulting cDNA was then used as a template for real-time PCR quantification. Real-time PCR was performed with 1/10 of the cDNA with specific primers for the quantification of IFN-α/β, and GAPDH gene expression with the iQ SYBR Green Supermix (Bio-Rad Laboratories, Hercules, CA) (Zhou et al., 2009, 2010). The oligonucleotide primers were synthesized by Integrated DNA Technologies, Inc. (Coralville, IA). The specific oligonucleotide primers used wereas follows: IFN-α:5′-TTTCTCCTGCCTGAAGGAC-AGAG-3′ (sense) and 5′-GCTCATGATTTCTGCTCTGACA-3′ (antisense) (Tsutsumi et al., 1999); IFN-β: 5′-GCCGCATTGACCATCTATGAGA-3′ (sense) and 5′-GAGATCTTCAGTTTCGGAGG-TAAC-3′ (antisense); GAPDH (the house keeping gene):5′- GGTGGTCTCCTCTGACTTCAACA-3′ (sense) and 5′-GTTGCTGTAGCCAAATTCGTTGT-3′ (anti-sense). The IFN-α primer pair matches 11 IFN-α subtypes: IFNA2, IFNA4, IFNA5, IFNA6, IFNA7, IFNA8, IFNA10, IFNA14, IFNA16, IFNA17, and IFNA21.

### HIV Strains and Other Reagents

Based on their differential use of the major HIV receptors (CCR5 and CXCR4), HIV isolates can be classified to R5, X4, and R5X4 strains (Berger et al., 1998). HIV Bal and JRFL strains (R5 tropic) were obtained from the AIDS Research and Reference Reagent Program at the NIH (Bethesda, MD, USA). HIV JAGO strain (R5 tropic) was obtained from the Center for AIDS Research at the School of Medicine, University of Pennsylvania. Heroin was kindly provided by Dr. Martin Adler from Temple University, Center for Substance Abuse Research through the NIDA drug supply program. Naltrexone was obtained from Sigma (St Louis, MO, USA).

### Heroin and/or Naltrexone Treatment

Seven-days-cultured macrophages (10^5^ cells/well in 96-well plates, or 2.5 × 10^5^ cells/well in 48-well plates) with or without heroin were incubated at different concentrations (10^-9^M to 10^-6^M) for 6 h. The selection of these concentrations of heroin was based on studies by others (Wang et al., 2002; Reynolds et al., 2006) and us (Guo et al., 2002; Wang et al., 2005, 2012). To investigate whether naltrexone blocks the heroin action, we treated the macrophages for 1 h with naltrexone (10^-6^M), followed by heroin treatment.

### Infection of Macrophages with HIV

Seven-days-cultured macrophages were infected with equal amounts of cell-free HIV Bal, JAGO, or JRFL strain for 2 h at 37°C after treatment with or without heroin. Cells were then washed three times with plain DMEM, to remove any unabsorbed virus, and fresh media containing heroin and/or naltrexone was added to the cell culture. Untreated cell served as a control. Culture supernatant was harvested at day 6, 10, and 12 postinfection for RT assay.

### HIV RT Assay

HIV RT activity was determined based on the technique (Willey et al., 1988) with modifications (Ho et al., 1992). In brief, 10 μl of culture supernatant from macrophages infected with or without HIV was added to a cocktail containing poly(A), oligo(dT) (Amersham Biosciences, Inc., Piscataway, NJ, USA), MgCl_2_, and [32P]dTTP (Amersham Biosciences, Inc.), and incubated for 24 h at 37°C. 30 μl of the cocktail was spotted onto DE81 paper (Whatman Internatianl Ltd, England), then dried and washed five times with 2 x saline-sodium citrate buffer and once with 95% ethanol. The filter paper was then air-dried. Radioactivity was counted in a liquid scintillation counter (PerkinElmer Life Sciences, Boston, MA, USA).

### Statistical Analysis

A student’s *t*-test was used to evaluate the significance of the differences between groups, and multiple comparisons were performed by regression analysis and one-way analysis of variance. Statistical analyses were performed with Graphpad Instat Statistical Software (Graphpad Software Inc., San Diego, CA, USA), and all data is presented as the mean ± SD. Statistical significance was defined as *P* < 0.05.

## Results

### Heroin Enhances HIV Infection of Macrophages

**FIGURE 1:**
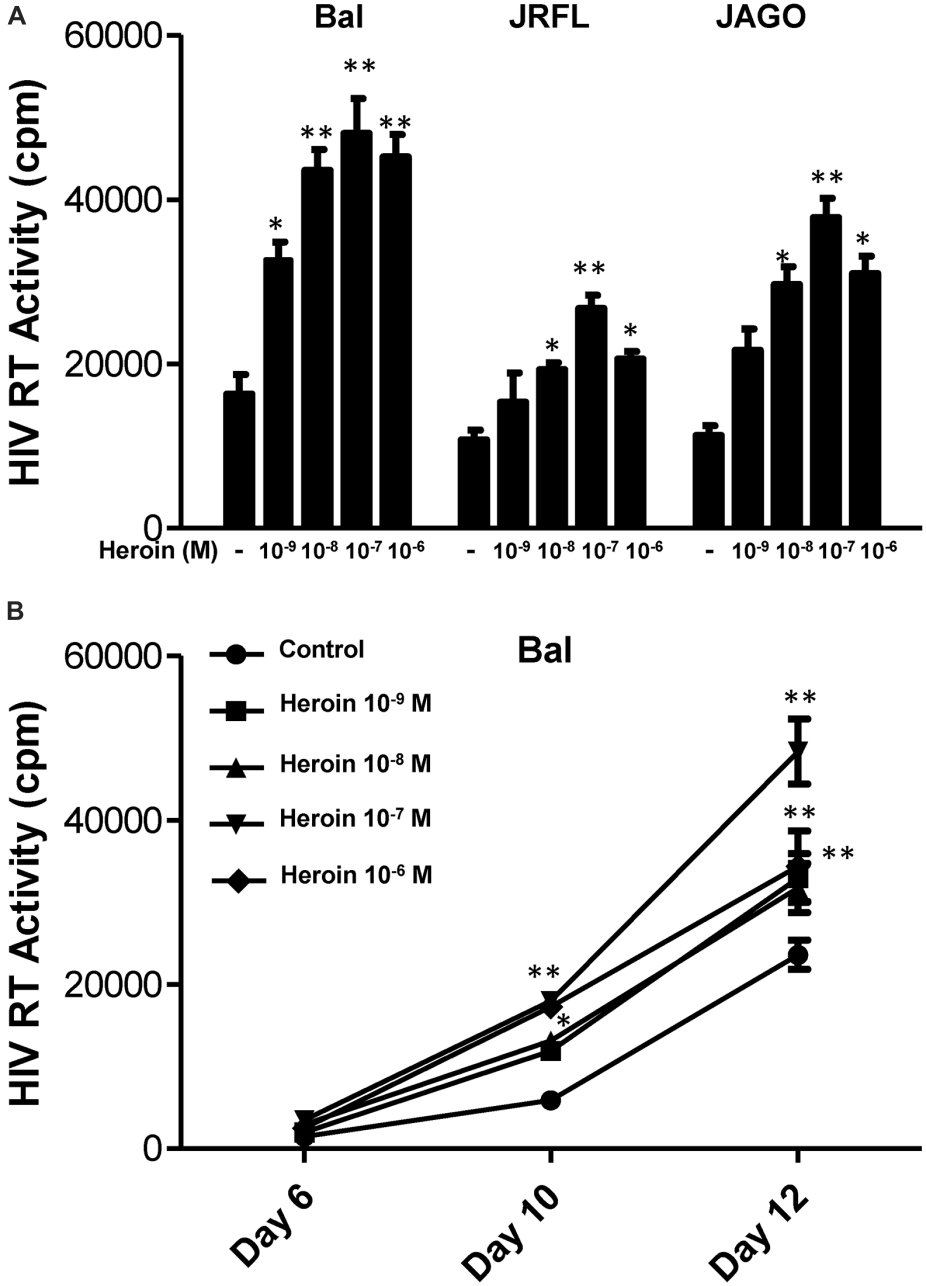
**Heroin enhances HIV infection of macrophages.** **(A)** Seven-day cultured macrophages were incubated with or without heroin at indicated concentrations for 6 h prior to different HIV strain (Bal, JRFL, or JAGO) infection. HIV RT activity was determined at day 12 postinfection. **(B)** Seven day cultured macrophages were incubated with or without heroin at the indicated concentrations for 6 h prior to HIV (Bal) infection. HIV RT activity was determined at day 6, 10, and 12 postinfection. Cell culture supernatant was collected at the indicated time points and subjected to RT assay to detect HIV RT activity. The data shown is the mean ± SD of triplicate cultures representative of three experiments using cells from three different donors (^∗^*p* < 0.05, ^∗∗^*p* < 0.01, heroin vs. control).

**FIGURE 2:**
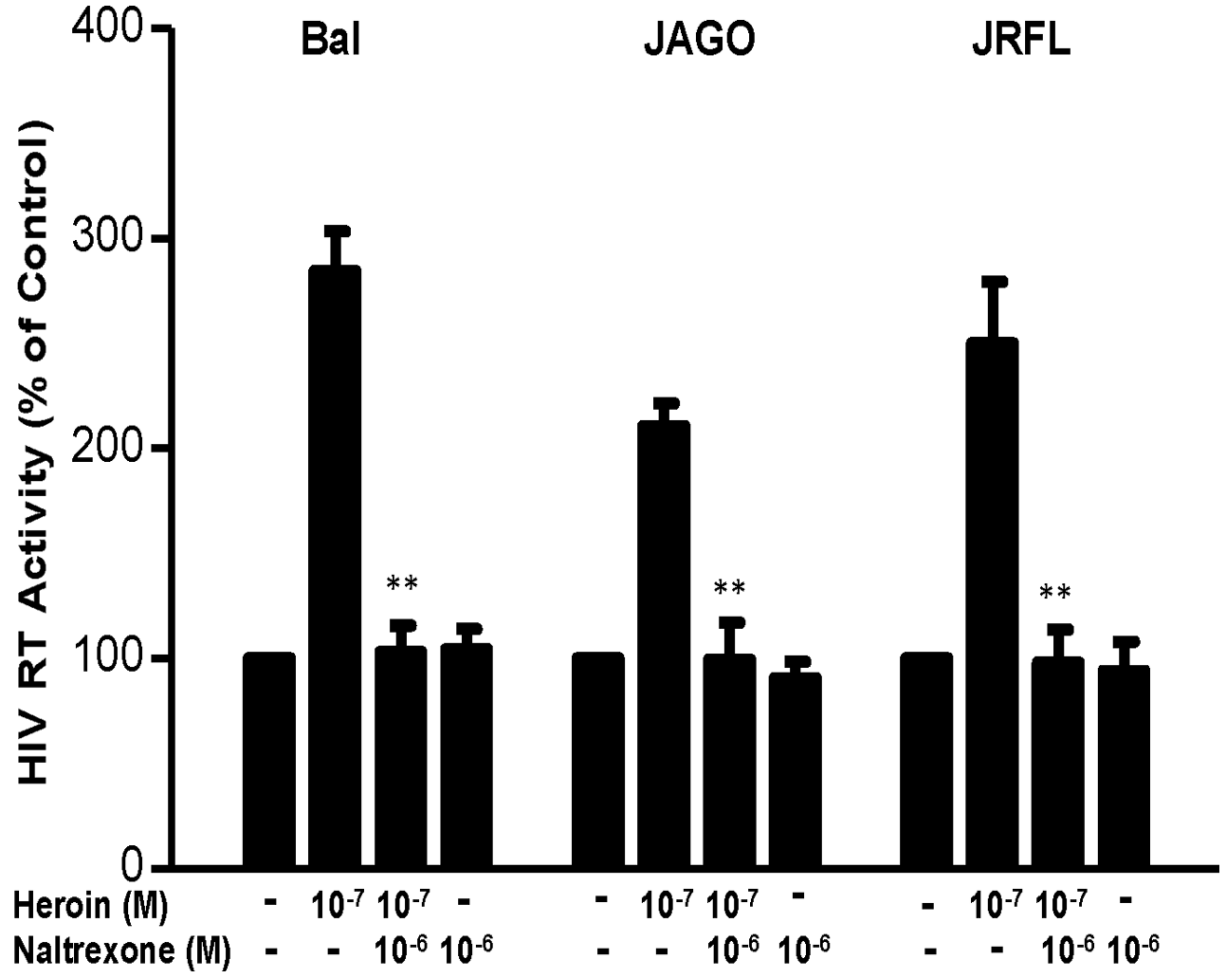
**Naltrexone blocks the heroin-mediated enhancement of HIV.** Seven-day cultured macrophages were incubated with or without heroin at the indicated concentrations for 6 h prior to HIV Bal, JRFL, or JAGO infection. Naltrexone (10^-6^ M) was added to the macrophages cultures for 1 h prior to heroin (10^-7^ M) treatment. HIV RT activity was determined at day 12 postinfection. Cell culture supernatant was collected at the indicated time points and subjected to RT assay to detect the HIV RT activity. The data shown is the mean ± SD of triplicate cultures representative of three experiments using cells from three different donors (^∗∗^*p* < 0.01, heroin vs. heroin + naltrexone).

We first determined the dose effect of heroin on HIV infection of macrophages. Heroin treatment enhanced the susceptibility of macrophages to infection with HIV Bal, JAGO, and JRFL strains, as evidenced by increased RT activity at day 12 postinfection (**Figure 1A**). We also examined the time course effect of heroin treatment on HIV infection of macrophages. As demonstrated in **Figure 1B**, heroin treatment increased HIV RT activity at different time points post infection. We further examined the effect of an opioid receptor antagonist on heroin-mediated enhancement of HIV RT activity. The pretreatment of macrophages with the opioid receptor antagonist (naltrexone) completely abrogated the enhancing effect of heroin on HIV RT activity (**Figure 2**).

### Heroin Inhibits HIV Restriction miRNAs

**FIGURE 3:**
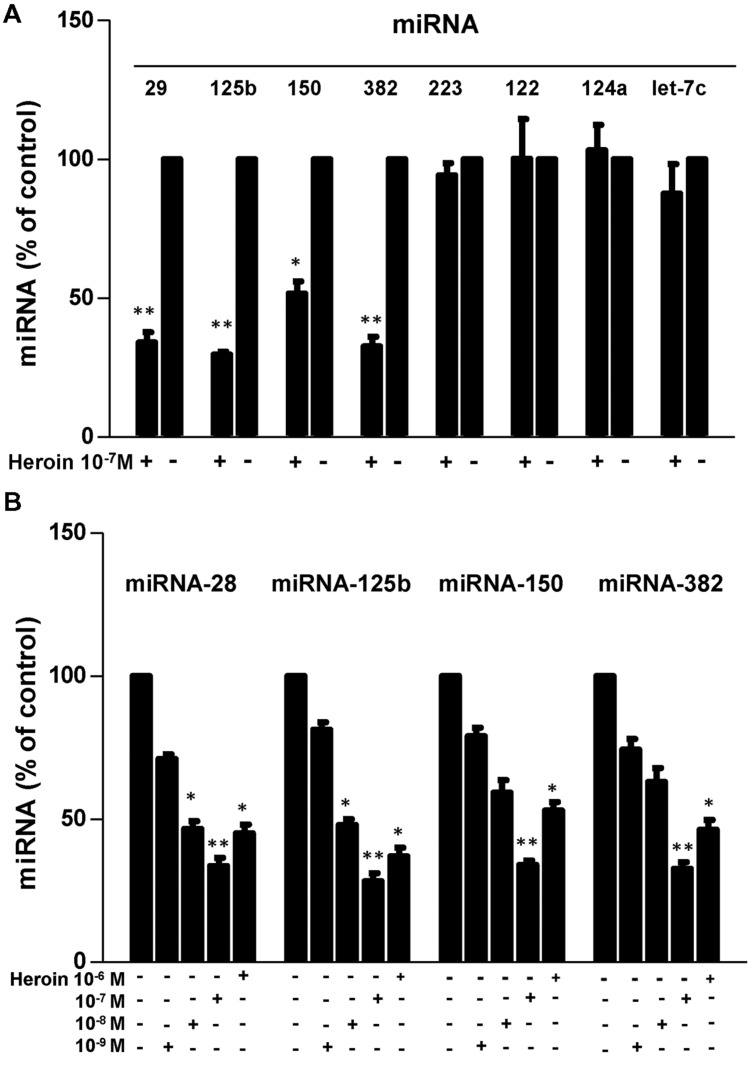
**Heroin inhibits HIV restriction miRNAs.** **(A)** Effect of heroin on miRNA expression in macrophages. Seven-day cultured macrophages were cultured in the presence or absence of heroin at the indicated concentration (10^-7^ M) for 6 h. Total cellular RNA was then extracted from cell cultures and subjected to real-time RT-PCR for miRNA-28, miRNA-125b, miRNA-150, miRNA-382, miRNA-223, miRNA-122, miRNA-124a, and Let-7c expression. Data is given as a mean ± SD of triplicate cultures representative of three experiments using cells from three different donors (^∗^*P* < 0.05, ^∗∗^*P* < 0.01 for heroin vs. control). **(B)** Seven-day cultured macrophages were incubated in the presence or absence of heroin at the indicated concentrations for 6 h. Total cellular RNA extracted from cell cultures was subjected to realtime RT-PCR for miRNA expression, miRNA-28, miRNA-125b, miRNA-150, and miRNA-382. The data shown is the mean ± SD of triplicate cultures representative of three experiments using cells from three different donors (^∗^*p* < 0.05, ^∗∗^*p* < 0.01, heroin vs. control).

**FIGURE 4:**
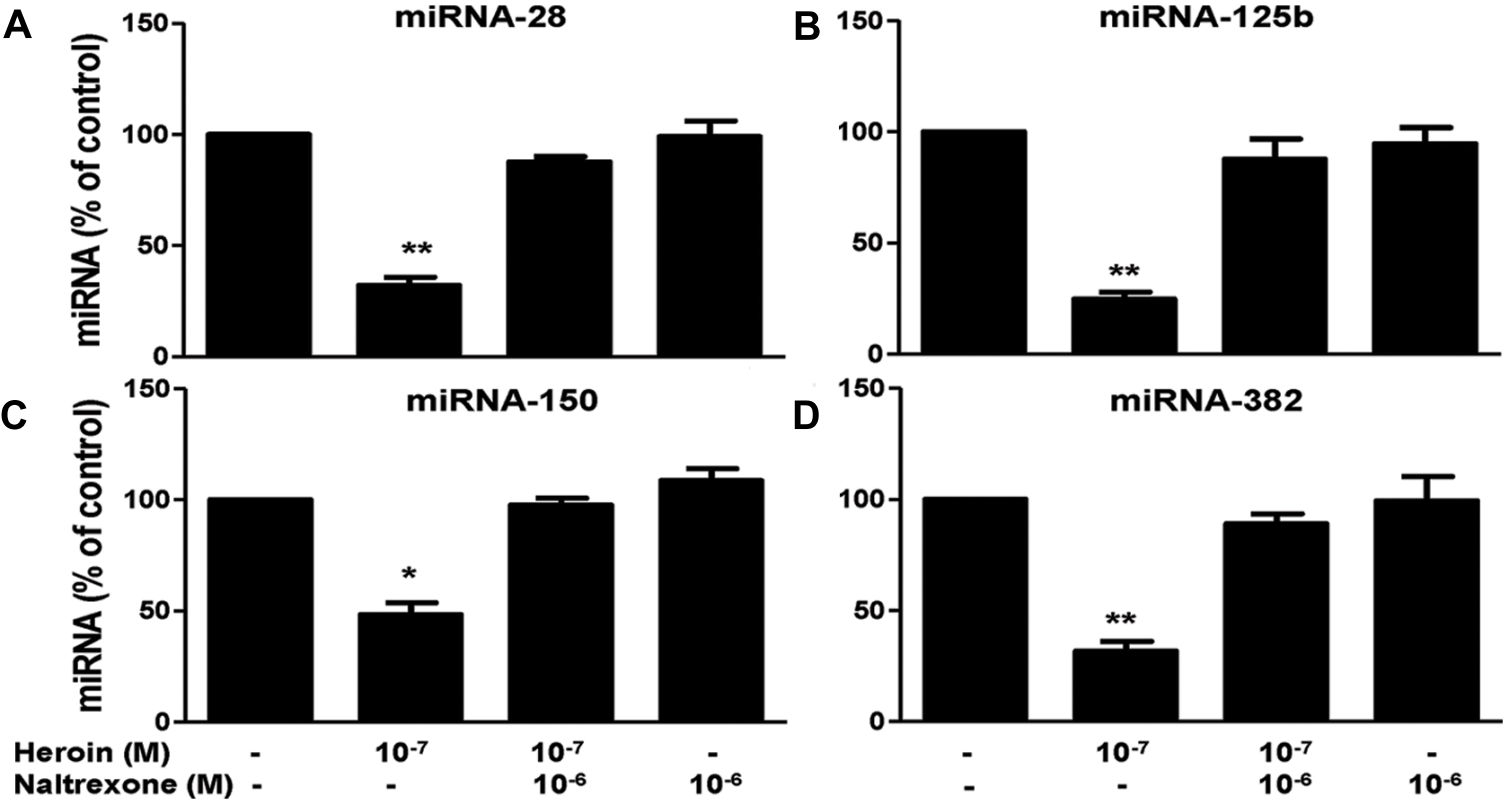
**Naltrexone blocks the heroin action on HIV restriction miRNAs.** Naltrexone (10^-6^ M) was added to macrophage cultures for 1 h prior to heroin treatment. Total cellular RNA was then extracted from the cell cultures 6 h post-treatment and subjected to realtime RT-PCR for miRNA expression: miRNA-28 **(A)**, miRNA-125b **(B)**, miRNA-150 **(C)**, and miRNA-382 **(D)**. The data shown is the mean ± SD of triplicate cultures representative of three experiments using cells from three different donors (^∗^*p* < 0.05, ^∗∗^*p* < 0.01, heroin vs. control, or heroin + naltrexone).

We first examined whether heroin has the ability to suppress the HIV restriction miRNAs in macrophages. As shown in **Figure 3**, heroin treatment of macrophages inhibited the expression of four HIV restriction miRNAs (miRNA-28, miRNA-125b, miRNA-150, and miRNA-382). The highest inhibition by heroin was observed at a concentration of 10^-7^M (**Figure 3B**). In contrast, heroin treatment of macrophages had little effect on the expression of miRNA-223, miRNA-124a, Let-7c, and miRNA-122 (**Figure 3A**). Since heroin-mediated enhancement of HIV infection could be blocked by naltrexone (**Figure 2**), we examined the effect of naltrexone on heroin-mediated inhibition of anti-HIV factor expression. As shown in **Figure 4**, naltrexone completely abrogated the suppressing effect of heroin on anti-HIV miRNA expression, whereas naltrexone alone had little effect on anti-HIV miRNA expression (**Figure 4**).

### Heroin Suppresses Type I IFNs

**FIGURE 5:**
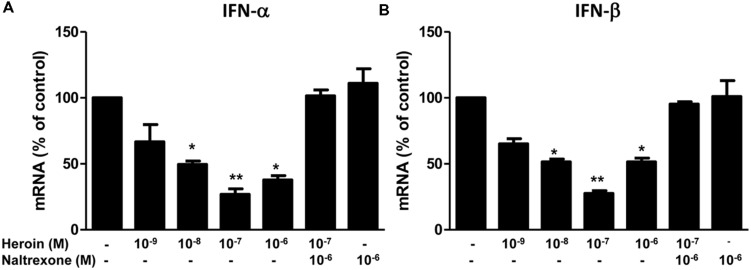
**Heroin inhibits IFN-α and IFN-β.** Naltrexone (10^-6^ M) was added to the macrophage cultures for 1 h prior to heroin treatment. Total cellular RNA was then extracted from the cell cultures 6 h post-treatment and subjected to realtime RT-PCR for IFN-α **(A)** and IFN-β **(B)** expression. The data shown is the mean ± SD of triplicate cultures representative of three experiments using cells from three different donors (^∗^*p* < 0.05, ^∗∗^*p* < 0.01, heroin vs. control, or heroin + naltrexone).

Type I IFNs (IFN-α/β) are well known for their ability to inhibit a wide range of virus(s), including HIV. Although the anti-HIV mechanism(s) of IFN-α/β remain to be determined, many cellular factors including miRNAs have been identified as IFN-α/β-inducible anti-HIV elements in target cells (Alter, 1999; Samuel, 2001; Sen, 2001; Okumura et al., 2006; Peng et al., 2006; Huang et al., 2007; Mosoian et al., 2007; Neil et al., 2008; Randall and Goodbourn, 2008; Van Damme et al., 2008; Wang et al., 2008). We thus examined whether heroin can suppress intracellular IFN-α and IFN-β expression in macrophages. As shown in **Figures 5A,B**, heroin treatment of macrophages, significantly suppressed IFN-α and IFN-β expression. The pretreatment of macrophages with naltrexone could block the effect of heroin on IFN-α and IFN-β expression (**Figures 5A,B**).

### *In Vivo* Impact of Heroin on HIV Restriction miRNAs

**FIGURE 6:**
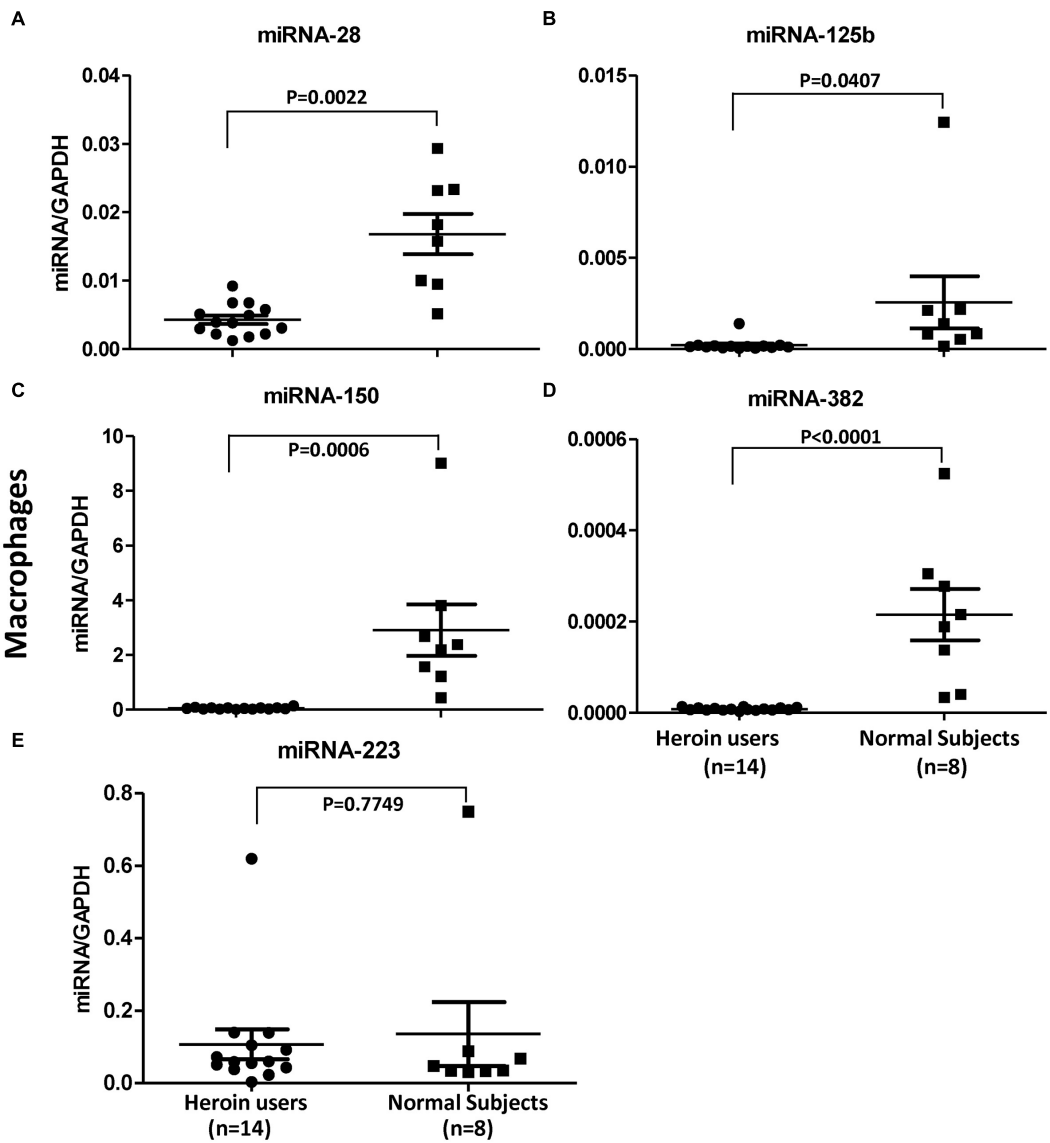
**Heroin users have decreased levels of HIV restriction miRNAs.** Total RNA was exacted from macrophages derived from monocytes of heroin addicts and normal control subjects. miRNA-28 **(A)**, miRNA-125b **(B)**, miRNA-150 **(C)**, miRNA-382 **(D)**, and miRNA-223 **(E)** was amplified using specific primers and quantified by real time RT-PCR. The results are expressed as relative transcript abundance of miRNA/GAPDH.

In order to confirm the *in vitro* action of heroin on the HIV restriction miRNAs, we examined the levels of these miRNAs in macrophages derived from monocytes of the heroin addicts and control subjects. As shown in **Figure 6**, heroin addicts had significantly lower levels of the HIV restriction miRNAs (miRNA-28, miRNA-125b, miRNA-150, and miRNA-382) than the control subjects. However, there was little difference in miRNA-223 levels between heroin addicts and control subjects (**Figure 6**).

## Discussion

Injection of heroin use increases the risk of acquiring HIV (Friedman et al., 2003; Sagar et al., 2015) and progression to AIDS (Ronald et al., 1994). However, because of the complexity of opioid addiction and/or HIV infection, it has been extremely difficult to compare different clinical and epidemiological findings in studying the impact of opioids on HIV disease progression (Donahoe et al., 2009). The majority of *in vitro* studies have shown that morphine enhances HIV infection of PBMC (Suzuki et al., 2002), CD4^+^ T cells (Chuang et al., 1993; Steele et al., 2003; Peterson et al., 2004), and macrophages (Guo et al., 2002; Li et al., 2002, 2003; Ho et al., 2003; Wang et al., 2011a, 2012). In the present study, we demonstrated for the first time that *in vitro* heroin treatment of macrophages increases HIV infection/replication with different strains (**Figure 1**). This effect of heroin on HIV was specific, as naltrexone could completely block the heroin action (**Figure 2**). We reported that human immune cells, including monocytes/macrophages, express functional μ opioid receptors (Guo et al., 2002; Ho et al., 2003; Wang et al., 2011a).

To understand the mechanism(s) of heroin action on HIV, we examined whether heroin use has a negative impact on the intracellular HIV-associated miRNAs. It is now known that a number of cellular miRNAs participate in host cell innate immunity against viral infections, including HIV. We were particularly interested in miRNA-28, miRNA-125b, miRNA-150, miRNA-223, and miR-382, as they can target the HIV RNA 3-terminus (a highly conserved region of the virus), inhibiting the translation of almost all HIV encoded proteins, including Rev and Tat, the key players in HIV replication in CD4^+^ T cells and macrophages (Hariharan et al., 2005). These miRNAs are enriched in resting CD4^+^ T cells (Huang et al., 2007) and monocytes (Wang et al., 2009), which contribute to the intracellular immune defense against HIV. The finding that heroin could inhibit the expression of the HIV restriction miRNAs provides a plausible mechanism for heroin-mediated enhancement of HIV infection of macrophages. Interestingly, heroin appears to selectively inhibit the HIV restriction miRNAs. We found that the inhibitory effect of heroin was limited to four of these miRNAs (miRNA-28, -125b, -150, and -382), while miR-223 and miRNA-124a/Let-7c were not affected by heroin treatment (**Figure 3A**). miRNA-124a/let-7c is HIV replication required miRNAs (Farberov et al., 2015), and miRNA-122 is a HCV-supportive miRNA (Fukuhara et al., 2012; Masaki et al., 2015).

These *in vitro* observations (**Figures 3** and **4**) were confirmed in our *in vivo* investigation, showing that macrophages derived from monocytes of the heroin addicts had lower levels of four HIV restriction miRNAs (miRNA-28, -125b, 150, and 382) than those from the control subjects. Similarly, heroin use had little effect on miRNA-223 expression as compared to the control groups (**Figure 6**). This observation could be due to the fact that miRNA-223 is not an inducible miRNA by IFN-α/β. We reported (Zhou et al., 2010) that although IFN-α/β could induce the expression of the HIV restriction miRNAs (miRNA-28, miRNA-125b, miRNA-150, and miRNA-382), they had little effect on miRNA-223 expression in macrophages.

The mechanism(s) of the heroin action on the HIV restriction miRNAs remains to be determined. However, it is likely that heroin-mediated suppression of type I IFNs is partially responsible for the heroin action on the miRNAs. As a potent inducer of antiviral activities, IFN-α/β plays a crucial role in host cell innate immunity. IFN-α/β can directly induce the expression of miRNAs that have antiviral activity. Our earlier study demonstrated that IFN-α/β treatment of macrophages induced the expression of the HIV restriction miRNAs (Zhou et al., 2010). We also reported that morphine treatment of monocytes could inhibit the expression of several HIV restriction miRNAs (Wang et al., 2011a). Studies by others showed that morphine suppressed IFN-α expression in PBMCs and T lymphocytes (Nair et al., 1997; Homan et al., 2002). In addition, morphine suppresses Sendai virus-induced IFN-α production by PBMCs (Nair et al., 1997). These findings support our observation that heroin could downregulate IFN-α/β expression in macrophages (**Figure 5**).

## Conclusion

Our study provides the compelling evidence that heroin enhances HIV replication in macrophages through the inhibition of the intracellular HIV restriction miRNAs. These findings support the notion that opioids compromise specific host innate defense mechanisms against HIV infection. Understanding how heroin abuse impairs specific immune responses to HIV infection should improve and advance our ability to treat HIV-infected heroin users. Nevertheless, future studies are necessary to reveal additional and unidentified mechanisms by which heroin damages the intracellular innate immunity that controls HIV in CD4^+^ T cells and macrophages.

## Conflict of Interest Statement

The authors declare that the research was conducted in the absence of any commercial or financial relationships that could be construed as a potential conflict of interest.

## References

[B1] Ahluwalia J. K. Khan S. Z. Soni K. Rawat P. Gupta A. Hariharan M. (2008). Human cellular microRNA hsa-miR-29a interferes with viral nef protein expression and HIV-1 replication. *Retrovirology* 5 117 10.1186/1742-4690-5-117 PMC2635386 19102781

[B2] Alcabes P. Friedland G. (1995). Injection drug use and human immunodeficiency virus infection. *Clin. Infect. Dis.* 20 1467–1479. 10.1093/clinids/20.6.1467 7548494

[B3] Alter M. J. (1999). Hepatitis C virus infection in the United States. *J. Hepatol.* 31(Suppl. 1), 88–91. 10.1016/S0168-8278(99)80381-X 10622567

[B4] Berger E. A. Doms R. W. Fenyo E. M. Korber B. T. Littman D. R. Moore J. P. (1998). A new classification for HIV-1. *Nature* 391 240 10.1038/35091 9440686

[B5] Boland J. W. McWilliams K. Ahmedzai S. H. Pockley A. G. (2014). Effects of opioids on immunologic parameters that are relevant to anti-tumour immune potential in patients with cancer: a systematic literature review. *Br. J. Cancer* 111 866–873. 10.1038/bjc.2014.384 25025960 PMC4150281

[B6] Brack A. Rittner H. L. Stein C. (2011). Immunosuppressive effects of opioids–clinical relevance. *J. Neuroimmune Pharmacol.* 6 490–502. 10.1007/s11481-011-9290-7 21728033

[B7] Chao C. C. Gekker G. Sheng W. S. Hu S. Portoghese P. S. Peterson P. K. (1995). Endogenous opioid peptides suppress cytokine-mediated upregulation of HIV-1 expression in the chronically infected promonocyte clone U1. *Adv. Exp. Med. Biol.* 373 65–72. 10.1007/978-1-4615-1951-5_10 7668162

[B8] Chen A. K. Sengupta P. Waki K. Van Engelenburg S. B. Ochiya T. Ablan S. D. (2014). MicroRNA binding to the HIV-1 Gag protein inhibits Gag assembly and virus production. *Proc. Natl. Acad. Sci. U.S.A.* 111 E2676–E2683. 10.1073/pnas.1408037111 24938790 PMC4084429

[B9] Chuang L. F. Killam K. F. Jr. Chuang R. Y. (1993). Increased replication of simian immunodeficiency virus in CEM x174 cells by morphine sulfate. *Biochem. Biophys. Res. Commun.* 195 1165–1173. 10.1006/bbrc.1993.2167 8216245

[B10] Cobos Jimenez V. Booiman T. de Taeye S. W. van Dort K. A. Rits M. A. Hamann J. (2012). Differential expression of HIV-1 interfering factors in monocyte-derived macrophages stimulated with polarizing cytokines or interferons. *Sci. Rep.* 2 763 10.1038/srep00763 PMC3478582 23094138

[B11] Donahoe R. M. Byrd L. D. McClure H. M. Fultz P. Brantley M. Marsteller F. (1993). Consequences of opiate-dependency in a monkey model of AIDS. *Adv. Exp. Med. Biol.* 335 21–28. 10.1007/978-1-4615-2980-4_4 8237597

[B12] Donahoe R. M. O’Neil S. P. Marsteller F. A. Novembre F. J. Anderson D. C. Lankford-Turner P. (2009). Probable deceleration of progression of Simian AIDS affected by opiate dependency: studies with a rhesus macaque/SIVsmm9 model. *J. Acquir. Immune Defic. Syndr.* 50 241–249. 10.1097/QAI.0b013e3181967354 19194320

[B13] Eisenstein T. K. (2011). Opioids and the immune system: what is their mechanism of action? *Br. J. Pharmacol.* 164 1826–1828. 10.1111/j.1476-5381.2011.01513.x 21627636 PMC3246707

[B14] Farberov L. Herzig E. Modai S. Isakov O. Hizi A. Shomron N. (2015). MicroRNA-mediated regulation of p21 and TASK1 cellular restriction factors enhances HIV-1 infection. *J. Cell Sci.* 128 1607–1616. 10.1242/jcs.167817 25717002 PMC4406127

[B15] Friedman H. Newton C. Klein T. W. (2003). Microbial infections, immunomodulation, and drugs of abuse. *Clin. Microbiol. Rev.* 16 209–219. 10.1128/CMR.16.2.209-219.2003 12692094 PMC153143

[B16] Fukuhara T. Kambara H. Shiokawa M. Ono C. Katoh H. Morita E. (2012). Expression of microRNA miR-122 facilitates an efficient replication in nonhepatic cells upon infection with hepatitis C virus. *J. Virol.* 86 7918–7933. 10.1128/JVI.00567-12 22593164 PMC3421686

[B17] Guo C. J. Li Y. Tian S. Wang X. Douglas S. D. Ho W. Z. (2002). Morphine enhances HIV infection of human blood mononuclear phagocytes through modulation of beta-chemokines and CCR5 receptor. *J. Investig. Med.* 50 435–442. 10.1097/00042871-200211010-00027 PMC4037869 12425430

[B18] Hariharan M. Scaria V. Pillai B. Brahmachari S. K. (2005). Targets for human encoded microRNAs in HIV genes. *Biochem. Biophys. Res. Commun.* 337 1214–1218. 10.1016/j.bbrc.2005.09.183 16236258

[B19] Ho W. Z. Guo C. J. Yuan C. S. Douglas S. D. Moss J. (2003). Methylnaltrexone antagonizes opioid-mediated enhancement of HIV infection of human blood mononuclear phagocytes. *J. Pharmacol. Exp. Ther.* 307 1158–1162. 14560041 10.1124/jpet.103.056697 PMC4016816

[B20] Ho W. Z. Lioy J. Song L. Cutilli J. R. Polin R. A. Douglas S. D. (1992). Infection of cord blood monocyte-derived macrophages with human immunodeficiency virus type 1. *J. Virol.* 66 573–579. 1727500 10.1128/jvi.66.1.573-579.1992 PMC238319

[B21] Homan J. W. Steele A. D. Martinand-Mari C. Rogers T. J. Henderson E. E. Charubala R. (2002). Inhibition of morphine-potentiated HIV-1 replication in peripheral blood mononuclear cells with the nuclease-resistant 2-5A agonist analog, 2-5A(N6B). *J. Acquir. Immune Defic. Syndr.* 30 9–20. 10.1097/00042560-200205010-00002 12048358

[B22] Huang J. Wang F. Argyris E. Chen K. Liang Z. Tian H. (2007). Cellular microRNAs contribute to HIV-1 latency in resting primary CD4+ T lymphocytes. *Nat. Med.* 13 1241–1247. 10.1038/nm1639 17906637

[B23] Klase Z. Houzet L. Jeang K. T. (2012). MicroRNAs and HIV-1: complex interactions. *J. Biol. Chem.* 287 40884–40890. 10.1074/jbc.R112.415448 23043098 PMC3510792

[B24] Kumar A. (2011). MicroRNA in HCV infection and liver cancer. *Biochim. Biophys. Acta* 1809 694–699. 10.1016/j.bbagrm.2011.07.010 21821155

[B25] Li Y. Merrill J. D. Mooney K. Song L. Wang X. Guo C. J. (2003). Morphine enhances HIV infection of neonatal macrophages. *Pediatr. Res.* 54 282–288. 10.1203/01.PDR.0000074973.83826.4C 12736382 PMC4035124

[B26] Li Y. Wang X. Tian S. Guo C. J. Douglas S. D. Ho W. Z. (2002). Methadone enhances human immunodeficiency virus infection of human immune cells. *J. Infect. Dis.* 185 118–122. 10.1086/338011 11756991 PMC4009627

[B27] Lu L. Fang Y. Wang X. (2008). Drug abuse in China: past, present and future. *Cell. Mol. Neurobiol.* 28 479–490. 10.1007/s10571-007-9225-2 17990098 PMC11515005

[B28] Martin M. Vanichseni S. Suntharasamai P. Mock P. A. Griensven F. V. Pitisuttithum P. (2010). Drug use and the risk of HIV infection amongst injection drug users participating in an HIV vaccine trial in Bangkok, 1999–2003. *Int. J. Drug Policy* 21 296–301. 10.1016/j.drugpo.2009.12.002 20079620

[B29] Masaki T. Arend K. C. Li Y. Yamane D. McGivern D. R. Kato T. (2015). miR-122 stimulates hepatitis C virus RNA synthesis by altering the balance of viral RNAs engaged in replication versus translation. *Cell Host Microbe* 17 217–228. 10.1016/j.chom.2014.12.014 25662750 PMC4326553

[B30] Meijerink H. Indrati A. Soedarmo S. Utami F. de Jong C. A. Alisjahbana B. (2015). Heroin use in Indonesia is associated with higher expression of CCR5 on CD4+ cells and lower ex-vivo production of CCR5 ligands. *AIDS* 29 385–388. 25834861

[B31] Mosoian A. Teixeira A. Burns C. S. Khitrov G. Zhang W. Gusella L. (2007). Influence of prothymosin-alpha on HIV-1 target cells. *Ann. N. Y. Acad. Sci.* 1112 269–285. 10.1196/annals.1415.043 17600282

[B32] Nair M. P. Schwartz S. A. Polasani R. Hou J. Sweet A. Chadha K. C. (1997). Immunoregulatory effects of morphine on human lymphocytes. *Clin. Diagn. Lab. Immunol.* 4 127–132. 9067644 10.1128/cdli.4.2.127-132.1997 PMC170490

[B33] Nathans R. Chu C. Y. Serquina A. K. Lu C. C. Cao H. Rana T. M. (2009). Cellular microRNA and P bodies modulate host-HIV-1 interactions. *Mol. Cell* 34 696–709. 10.1016/j.molcel.2009.06.003 19560422 PMC2763548

[B34] Neil S. J. Zang T. Bieniasz P. D. (2008). Tetherin inhibits retrovirus release and is antagonized by HIV-1 Vpu. *Nature* 451 425–430. 10.1038/nature06553 18200009

[B35] Okumura A. Lu G. Pitha-Rowe I. Pitha P. M. (2006). Innate antiviral response targets HIV-1 release by the induction of ubiquitin-like protein ISG15. *Proc. Natl. Acad. Sci. U.S.A.* 103 1440–1445. 10.1073/pnas.0510518103 16434471 PMC1360585

[B36] Pedersen I. M. Cheng G. Wieland S. Volinia S. Croce C. M. Chisari F. V. (2007). Interferon modulation of cellular microRNAs as an antiviral mechanism. *Nature* 449 919–922. 10.1038/nature06205 17943132 PMC2748825

[B37] Peng G. Lei K. J. Jin W. Greenwell-Wild T. Wahl S. M. (2006). Induction of APOBEC3 family proteins, a defensive maneuver underlying interferon-induced anti-HIV-1 activity. *J. Exp. Med.* 203 41–46. 10.1084/jem.20051512 16418394 PMC2118075

[B38] Peterson P. K. Gekker G. Brummitt C. Pentel P. Bullock M. Simpson M. (1989). Suppression of human peripheral blood mononuclear cell function by methadone and morphine. *J. Infect. Dis.* 159 480–487. 10.1093/infdis/159.3.480 2536791

[B39] Peterson P. K. Gekker G. Hu S. Cabral G. Lokensgard J. R. (2004). Cannabinoids and morphine differentially affect HIV-1 expression in CD4(+) lymphocyte and microglial cell cultures. *J. Neuroimmunol.* 147 123–126. 10.1016/j.jneuroim.2003.10.026 14741442

[B40] Peterson P. K. Gekker G. Hu S. Lokensgard J. Portoghese P. S. Chao C. C. (1999). Endomorphin-1 potentiates HIV-1 expression in human brain cell cultures: implication of an atypical mu-opioid receptor. *Neuropharmacology* 38 273–278. 10.1016/S0028-3908(98)00167-1 10218868

[B41] Pomorska D. K. Gach K. Janecka A. (2014). Immunomodulatory effects of endogenous and synthetic peptides activating opioid receptors. *Mini Rev. Med. Chem.* 14 1148–1155. 25553430 10.2174/1389557515666150101095237

[B42] Randall R. E. Goodbourn S. (2008). Interferons and viruses: an interplay between induction, signalling, antiviral responses and virus countermeasures. *J. Gen. Virol.* 89 1–47. 10.1099/vir.0.83391-0 18089727

[B43] Reynolds J. L. Mahajan S. D. Sykes D. Nair M. P. (2006). Heroin-induces differential protein expression by normal human astrocytes (NHA). *Am. J. Infect. Dis.* 2 49–57. 10.3844/ajidsp.2006.49.57 17235376 PMC1775911

[B44] Risdahl J. M. Khanna K. V. Peterson P. K. Molitor T. W. (1998). Opiates and infection. *J. Neuroimmunol.* 83 4–18. 10.1016/S0165-5728(97)00216-6 9610668

[B45] Ronald P. J. Robertson J. R. Elton R. A. (1994). Continued drug use and other cofactors for progression to AIDS among injecting drug users. *Aids* 8 339–343. 10.1097/00002030-199403000-00007 7913328

[B46] Sagar V. Pilakka-Kanthikeel S. Atluri V. S. Ding H. Arias A. Y. Jayant R. D. (2015). Therapeutical neurotargeting via magnetic nanocarrier: implications to opiate-induced neuropathogenesis and neuroAIDS. *J. Biomed. Nanotechnol.* 11 1–12. 10.1166/jbn.2015.2108 26502636 PMC4624254

[B47] Samikkannu T. Ranjith D. Rao K. V. Atluri V. S. Pimentel E. El-Hage N. (2015). HIV-1 gp120 and morphine induced oxidative stress: role in cell cycle regulation. *Front. Microbiol.* 6:614 10.3389/fmicb.2015.00614 PMC4477635 26157430

[B48] Samuel C. E. (2001). Antiviral actions of interferons. *Clin. Microbiol. Rev.* 14 778–809. 10.1128/CMR.14.4.778-809.2001 11585785 PMC89003

[B49] Schweitzer C. Keller F. Schmitt M. P. Jaeck D. Adloff M. Schmitt C. (1991). Morphine stimulates HIV replication in primary cultures of human Kupffer cells. *Res. Virol.* 142 189–195. 10.1016/0923-2516(91)90056-9 1896643

[B50] Sen G. C. (2001). Viruses and interferons. *Annu. Rev. Microbiol.* 55 255–281. 10.1146/annurev.micro.55.1.255 11544356

[B51] Specter S. (1994). Drugs of abuse and infectious diseases. *J. Fla. Med. Assoc.* 81 485–487. 7964576

[B52] Squinto S. P. Mondal D. Block A. L. Prakash O. (1990). Morphine-induced transactivation of HIV-1 LTR in human neuroblastoma cells. *AIDS Res. Hum. Retroviruses* 6 1163–1168. 10.1089/aid.1990.6.1163 2252636

[B53] Steele A. D. Henderson E. E. Rogers T. J. (2003). Mu-opioid modulation of HIV-1 coreceptor expression and HIV-1 replication. *Virology* 309 99–107. 10.1016/S0042-6822(03)00015-1 12726730

[B54] Sung T. L. Rice A. P. (2009). miR-198 inhibits HIV-1 gene expression and replication in monocytes and its mechanism of action appears to involve repression of cyclin T1. *PLoS Pathog.* 5:e1000263 10.1371/journal.ppat.1000263 PMC2607557 19148268

[B55] Suzuki S. Carlos M. P. Chuang L. F. Torres J. V. Doi R. H. Chuang R. Y. (2002). Methadone induces CCR5 and promotes AIDS virus infection. *FEBS Lett.* 519 173–177. 10.1016/S0014-5793(02)02746-1 12023039

[B56] Tsutsumi H. Takeuchi R. Ohsaki M. Seki K. Chiba S. (1999). Respiratory syncytial virus infection of human respiratory epithelial cells enhances inducible nitric oxide synthase gene expression. *J. Leukoc. Biol.* 66 99–104. 10410996

[B57] Van Damme N. Goff D. Katsura C. Jorgenson R. L. Mitchell R. Johnson M. C. (2008). The interferon-induced protein BST-2 restricts HIV-1 release and is downregulated from the cell surface by the viral Vpu protein. *Cell Host Microbe* 3 245–252. 10.1016/j.chom.2008.03.001 18342597 PMC2474773

[B58] Wang F. X. Huang J. Zhang H. Ma X. Zhang H. (2008). APOBEC3G upregulation by alpha interferon restricts human immunodeficiency virus type 1 infection in human peripheral plasmacytoid dendritic cells. *J. Gen. Virol.* 89 722–730. 10.1099/vir.0.83530-0 18272764

[B59] Wang J. Charboneau R. Balasubramanian S. Barke R. A. Loh H. H. Roy S. (2002). The immunosuppressive effects of chronic morphine treatment are partially dependent on corticosterone and mediated by the mu-opioid receptor. *J. Leukoc. Biol.* 71 782–790. 11994502

[B60] Wang X. Ho W. Z. (2011). Drugs of abuse and HIV infection/replication: implications for mother-fetus transmission. *Life Sci.* 88 972–979. 10.1016/j.lfs.2010.10.029 21056582 PMC3100448

[B61] Wang X. Tan N. Douglas S. D. Zhang T. Wang Y. J. Ho W. Z. (2005). Morphine inhibits CD8+ T cell-mediated, noncytolytic, anti-HIV activity in latently infected immune cells. *J. Leukoc. Biol.* 78 772–776. 10.1189/jlb.0305167 16000393

[B62] Wang X. Ye L. Hou W. Zhou Y. Wang Y. J. Metzger D. S. (2009). Cellular microRNA expression correlates with susceptibility of monocytes/macrophages to HIV-1 infection. *Blood* 113 671–674. 10.1182/blood-2008-09-175000 19015395 PMC2628373

[B63] Wang X. Ye L. Zhou Y. Liu M. Q. Zhou D. J. Ho W. Z. (2011a). Inhibition of anti-HIV microRNA expression: a mechanism for opioid-mediated enhancement of HIV infection of monocytes. *Am. J. Pathol.* 178 41–47. 10.1016/j.ajpath.2010.11.042 21224041 PMC3069926

[B64] Wang X. Zhang T. Ho W. Z. (2011b). Opioids and HIV/HCV infection. *J. Neuroimmune Pharmacol.* 6 477–489. 10.1007/s11481-011-9296-1 21755286 PMC3937260

[B65] Wang Y. Wang X. Ye L. Li J. Song L. Fulambarkar N. (2012). Morphine suppresses IFN signaling pathway and enhances AIDS virus infection. *PLoS ONE* 7:e31167 10.1371/journal.pone.0031167 PMC3281044 22359571

[B66] Willey R. L. Smith D. H. Lasky L. A. Theodore T. S. Earl P. L. Moss B. (1988). In vitro mutagenesis identifies a region within the envelope gene of the human immunodeficiency virus that is critical for infectivity. *J. Virol.* 62 139–147. 3257102 10.1128/jvi.62.1.139-147.1988 PMC250512

[B67] Zhou Y. Sun L. Wang X. Zhou L. Li J. Liu M. (2015). Heroin use promotes HCV infection and dysregulates HCV-related circulating microRNAs. *J. Neuroimmune Pharmacol.* 10 102–110. 10.1007/s11481-014-9577-6 25572448 PMC4444785

[B68] Zhou Y. Wang X. Liu M. Hu Q. Song L. Ye L. (2010). A critical function of toll-like receptor-3 in the induction of anti-human immunodeficiency virus activities in macrophages. *Immunology* 131 40–49. 10.1111/j.1365-2567.2010.03270.x 20636339 PMC2966756

[B69] Zhou Y. Ye L. Wan Q. Zhou L. Wang X. Li J. (2009). Activation of Toll-like receptors inhibits herpes simplex virus-1 infection of human neuronal cells. *J. Neurosci. Res.* 87 2916–2925. 10.1002/jnr.22110 19437550 PMC3935181

